# A Single-Centre Experience of Post-COVID-19 Vaccine-Related Immune-Mediated Complications

**DOI:** 10.1155/2022/4742639

**Published:** 2022-09-30

**Authors:** David Palmer, Lauren Davis, Helena Sivaloganathan, Timothy Chevassut

**Affiliations:** ^1^Royal Sussex County Hospitals, University Hospitals Sussex, Brighton BN2 5BE, UK; ^2^Brighton and Sussex Medical School, University of Sussex, Brighton BN1 9PS, UK

## Abstract

The significant impact of the COVID-19 pandemic has resulted in a worldwide effort to develop effective vaccines. In the United Kingdom, the COVID-19 vaccine development and roll-out has been overwhelmingly successful in reducing infections and deaths. However, case reports have emerged of a rare syndrome of vaccine-induced immune thrombocytopenia and thrombosis (VITT), as well as cases of immune thrombocytopenia (ITP). This has necessitated a better understanding of these conditions. However, as both VITT and “vaccine-associated ITP” are emerging conditions, evidence on the clinical features, epidemiology, and management is still evolving. Subsequently, with the initiation of the COVID-19 vaccine booster program, it has become increasingly important to continue to collect accurate data on post-COVID-19 vaccine complications to aid with their prompt recognition and management. In this case series, we report on the presentations and management of seven cases of post-COVID-19 vaccine-related immune-mediated complications which occurred at our center between the months of March and July 2021.

## 1. Introduction

Vaccine-induced immune thrombocytopenia and thrombosis, or VITT, is a potentially severe condition featuring a combination of thrombocytopenia, high D-dimer levels, low fibrinogen, and thrombotic events, occurring 5–30 days following a dose of the COVID-19 vaccine [1]. These thrombotic events include cerebral venous sinus thrombosis, pulmonary embolus, splanchnic vein thrombosis, and arterial thromboses and can be life threatening [1, 2]. It was initially characterized after the AstraZeneca vaccine (ChAdOx1 nCoV-19) [1, 3] though there have also been reports after the Johnson & Johnson (AD26.COV2.S) and potentially the mRNA Pfizer-BioNTech (BNT162b2) and the Moderna (mRNA1273) vaccines [1, 4, 5]. It appears to mimic HIT (heparin-induced thrombocytopenia) with the production of anti-PF4 antibodies [3]. Further evidence suggests that these thrombotic events can be more severe than thromboses occurring in the absence of VITT [6], but the clinical presentation is highly variable and overlaps with other syndromes more reminiscent of immune thrombocytopenia [7].

## 2. Cases

### 2.1. Case 1

**Figure 1:**
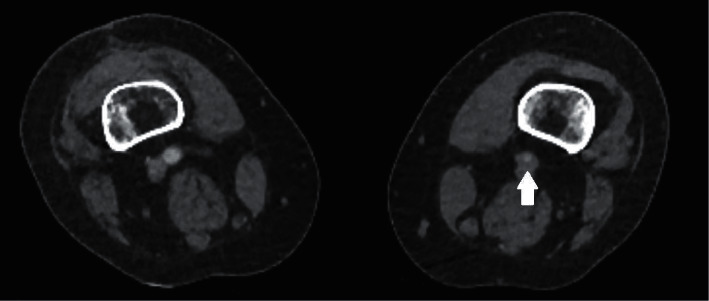
A CT angiogram demonstrates an occlusive thrombus (white arrow) of the left common femoral artery.

A 59-year-old woman with a past medical history of depression, hypertension, and hypothyroidism presented 19 days after her first dose of the AstraZeneca (ChAdOx1 nCoV-19) COVID-19 vaccine. Her initial symptoms were headaches, but she later developed severe pain and numbness in her left leg. On admission, she had a platelet count of 16 × 10^9^/L, D-dimer of 36.00 ug/ml, and fibrinogen level of 2.6 g/L. An APTT and PT time were within normal range. She underwent a CT angiogram of the leg which showed an acute thrombus of the left common femoral artery (Figure 1). She was treated with two repeated doses of intravenous immunoglobulin, IVIg (1 g/kg), over two days, was started on an argatroban infusion, and was given four days of dexamethasone (40 mg once daily). Anti-PF4 antibodies were detected at 0.285 (OD units). By day six, her platelet count had risen to 191 × 10^9^/L. However, her leg had become increasingly ischaemic and was nonviable. Subsequently, she underwent a transfemoral above-knee amputation. Postoperatively, she was switched to fondaparinux and was later commenced on aspirin and rivaroxaban. Repeat anti-PF4 antibodies four weeks later were no longer elevated. She subsequently received a dose of the Pfizer (BNT162b2) vaccine without any complications.

### 2.2. Case 2

A 58-year-old man with a past medical history of depression and gastritis presented 11 days after his first dose of the AstraZeneca (ChAdOx1 nCoV-19) COVID-19 vaccine. He had a three-day history of headaches and generalized myalgia. On admission, he had a trough platelet count of 20 × 10^9^/L, peak D-dimer of 54.30 ug/ml, and fibrinogen of 1.3 g/L. An APTT and PT time were within normal limits. He underwent a cerebral CT venogram and MR venogram which showed no evidence of a cerebral vein thrombus. An ultrasound scan of the abdomen showed no evidence of a splanchnic vein thrombus. Anti-PF4 antibodies were detected at 0.245 (OD units). He was given one dose of IVIg (1 mg/kg) and dexamethasone (40 mg once daily) for four days and started on the treatment dose of fondaparinux. By day seven, his platelet count had risen to 188 × 10^9^/L, and by week five, his D-dimer and anti-PF4 antibodies were no longer elevated (<0.28 ug/ml and 0.141 OD units, respectively). He was switched to a six-month course of rivaroxaban and later went on to have a dose of the Pfizer (BNT162b2) vaccine without any complications.

### 2.3. Case 3

A 61-year-old woman with well-controlled rheumatoid arthritis on sulfasalazine presented 25 days after her first dose of the AstraZeneca (ChAdOx1 nCoV-19) COVID-19 vaccine. Her presenting symptoms were of bruising over the extremities, bleeding from the mouth, and a petechial rash over the legs. On admission, she had a platelet count of 5 × 10^9^/L with an elevated D-dimer at 1.03 ug/ml. Anti-PF4 antibodies were detected at 0.247 (OD units). Her sulfasalazine was held. She initially had a poor response to IVIg (1 mg/kg) and four days of dexamethasone (40 mg once daily) which was switched at day six to 1 mg/kg of prednisolone when she received a further dose of IVIg. By day eight, her platelets had risen to over 50 × 10^9^/L. For a further six weeks, her platelet count was maintained between 50 and 150 × 10^9^/L and D-dimers were noted to fall to normal limits during this time. However, there was a subsequent fall in her platelet count shortly after this, associated again with rising D-dimer levels. She was subsequently commenced on eltrombopag and an improvement in her platelet count was sustained at over 50 × 10^9^/L, associated with falling D-dimers. She later went on to have a dose of the Pfizer (BNT162b2) vaccine without any complications. She continues on eltrombopag and a low dose of prednisolone.

### 2.4. Case 4

A 66-year-old man with a history of benign prostatic hyperplasia presented 34 days following the first dose of his Pfizer (BNT162b2) COVID-19 vaccine, with epistaxis and bruising. On admission, his platelet count was 1 × 10^9^/L and D-dimers were mildly elevated at 1.87 *u*g/ml. His anti-PF4 antibodies were positive at 0.285 OD units. He was given four days of dexamethasone (20 mg once daily) and IVIg (1 mg/kg). He had a good response to this treatment initially with platelets increasing to 108 × 10^9^/L on day five. However, following this, his platelets dropped resulting in him being recommenced on both a weaning regime of prednisolone and eltrombopag. He had a good response to these agents, and the eltrombopag was discontinued after eight weeks with the platelet count remaining above 100 × 10^9^/L. He was advised to get a second dose of either the AstraZeneca or Moderna COVID-19 vaccine.

### 2.5. Case 5

A 49-year-old woman with mild COPD presented 14 days after her first dose of the AstraZeneca (ChAdOx1 nCoV-19) COVID-19 vaccine. Her initial symptoms were bruising over the extremities and headaches. On admission, she had a platelet count of 26 × 10^9^/L and normal D-dimers. A CT venogram followed by an MR venogram showed no evidence of cerebral vein thrombus. Anti-PF4 antibodies were strongly positive at 0.734 (OD units). She was given four days of dexamethasone (20 mg once daily) and IVIg (1 mg/kg). By day eight, her platelets had responded to 300 × 10^9^/L. However, a week later they fell to less than 100 × 10^9^/L. She was subsequently started on a weaning regime of prednisolone at (1 mg/kg) for which an ongoing response continues. She later went on to have a dose of the Pfizer (BNT162b2) vaccine without any complications.

### 2.6. Case 6

A 42-year-old man with no significant medical history presented 12 days after his first dose of the AstraZeneca (ChAdO × 1 nCoV-19) COVID-19 vaccine. His initial symptoms were a spreading petechial rash over both legs. On admission, his platelet count was 16 × 10^9^/L and D-dimers were normal. Anti-PF4 antibodies were not elevated (0.0702 OD units). He was commenced on four days of dexamethasone (40 mg once daily) and given IVIg (1 mg/kg). By day seven, his platelet count had risen to 212 × 10^9^/L and remains within normal limits since. He has not yet had a dose of an alternative COVID-19 vaccine.

### 2.7. Case 7

A 49-year-old woman with no medical history presented 21 days after her first dose of the AstraZeneca (ChAdO × 1 nCoV-19) COVID-19 vaccine. Her initial symptoms were bruising, blisters in the mouth, and a petechial rash. On admission, her platelet count was 1 × 10^9^/L and D-dimers were normal. Anti-PF4 antibodies were not detected at elevated levels (0.172 OD units). She was given four days of dexamethasone (40 mg once daily) and IVIg (1 mg/kg). By day seven, there had been a limited response in her platelet count, remaining <10 × 10^9^/L; therefore, a repeat dose of IVIg (1 mg/kg) was given. By day 28, her platelet count had risen to 207 × 10^9^/L and has remained within a normal range since. She has not yet had a second dose of an alternative COVID-19 vaccine.

## 3. Discussion

Presentations of VITT have been described as an autoimmune-prothrombotic disorder phenotypically comparable to “spontaneous HIT,” but without any prior heparin exposure [8, 9]. In Case 1, based on the criteria set out by Pavord et al. [2], all five criteria had been met to satisfy a diagnosis of “definite VITT,” whereas Case 2 only met the criteria for “probable VITT.” Case 2 could be considered as “pre-VITT,” previously described as the full syndrome in the absence of thrombosis [10]. Subsequently, a “subclinical” presentation based on the biochemical presence of thrombocytopenia, raised D-dimers, and anti-platelet factor 4 antibodies could precede any thrombotic events. Thus, VITT could exist on a spectrum, not always fulfilling the defined criteria, as has been suggested [11]. The timely implementation of anticoagulation, in this case, is likely to have averted thrombosis, most notably cerebral venous sinus thrombosis, which could have resulted in significant consequences for this patient, as has been reported [10, 12, 13]. Theoretically, repeat vaccine doses could further stimulate these “subclinical” anti-PF4 antibody levels, increasing the risk of VITT with second/booster doses, which has been suggested [14]. However, most cases to date have occurred following the first vaccine doses. Importantly, screening tests for VITT have not been recommended [15].

Perhaps receiving less initial attention than VITT, “vaccine-associated ITP” has been recognized as a complication of both the AstraZeneca (ChAdO × 1 nCoV-19) and Pfizer (BNT162b2) vaccines[7, 16–18]. In our center, we encountered patients who presented with suspected “vaccine-associated ITP” following both the AstraZeneca (ChAdO × 1 nCoV-19) and Pfizer (BNT162b2) vaccines (cases 3 to 7). Notably, the clinical presentation of each of these cases was similar to the sequelae seen in usual ITP, most commonly with a petechial rash and mucocutaneous bleeding. However, in each case, we report the variable levels of D-dimers measured and anti-PF4 antibodies detected. This could suggest an overlap between VITT and “vaccine-associated ITP,” and whilst there remains a lack of understanding as to the different aetiological mechanisms, this heterogeneity of presentations might support the concept of a “spectrum” on which these conditions exist. The presentations, laboratory findings, management, and outcome have been summarised in Table 1.

### 3.1. The Role of Diagnostic Tests in VITT and “Vaccine-Associated ITP”

As outlined by Pavord et al. [2], anti-PF4 antibodies form a key part of the diagnosis of VITT. In parallel to HIT, these antibodies are proposed to be an important mechanism in the pathophysiology of thrombocytopenia and thrombosis seen in VITT. Anti-PF4 antibodies from patients with VITT bind a similar site to heparin on PF4, creating immune complexes that activate platelets via Fc*γ* receptor IIa [19]. In HIT, this activation contributes to a hypercoagulable state, followed by platelet death [20]. In this case series, the same ELISA method was used to detect anti-PF4 antibodies, making their results comparable. For the most part, we have seen some correlation between anti-PF4 optical density and severity of disease supporting a causal role of these antibodies, as evidenced by the higher anti-PF4 antibody levels in Case 1, with arterial thrombosis, compared with Case 2, where no clinical thrombosis was demonstrated. The key exception to this in our cases was Case 5 where anti-PF4 antibody levels were strongly positive at 0.734 OD units. This raises a number of questions regarding the significance of these antibodies in this case, and further understanding is needed on anti-PF4 antibodies and their binding kinetics in relation to coagulation and thrombogenesis. Whilst it is possible that the anti-PF4 antibodies in Case 5 were nonpathogenic, possibly due to nonspecific binding, it does suggest that VITT and “vaccine-associated ITP” exist upon a spectrum of diseases.

In the diagnosis of HIT, the lower specificity of anti-PF4 immunoassays is an important consideration, necessitating the use of clinical judgment and a pre-test probability score [20]. The criteria set out by Pavord et al. [2] are a step toward the development of a clinical assessment tool, to further rationalize the use of antibody tests. Subsequent analysis is required to ensure the sensitivity and specificity of any scoring system, particularly if the disease processes of VITT and “vaccine-associated ITP” are intrinsically linked. It is clear that no single test captures a diagnosis of VITT or “vaccine-associated ITP.” Instead, a constellation of biochemical tests and clinical observations are required, and it remains important that the emphasis is on early detection and management.

### 3.2. The Management of VITT and “Vaccine-Associated ITP” at Our Centre

The treatment of VITT includes the management of any thrombus and suppressing the immune response. For the initial management of the immune response, 1 g/kg of IVIg is advised [21]. If the patient is showing progression of thrombosis or the platelet count fails to rise, options include further doses of IVIg, corticosteroids, plasma exchange, and rituximab [21]. Alongside the medical management, the surgical treatment of thrombosis in VITT includes embolectomy/thrombectomy, fasciotomy, or amputation (which occurred in Case 1), as has been described in several other cases [22–25].

One patient (Case 2) was started on anticoagulation for pre-VITT/probable VITT (without evidence of thrombosis), as there was suspicion of a high risk of impending thrombosis from the laboratory results. This is in line with current NICE guidance [21], who advocates prophylactic anticoagulation for those with VITT without thrombosis, especially while anti-PF4 antibodies are still present. By contrast, some of the patients with “vaccine-associated ITP” had detectable anti-PF4 antibodies, but as they primarily presented with a bleeding phenotype, they were not given anticoagulation. It is unclear moving forward whether these patients, especially those with detectable anti-PF4 antibodies, remain at risk of thrombosis.

Patients with VITT require ongoing follow-up, both initially with frequent blood tests (every 2–3 days for the first weeks) [21] and in a specialist hematology clinic. This will allow patients not only to be cared for in their recovery but also to be monitored for biochemical signs of relapse. None of the cases of VITT in this series had further episodes of thrombosis or thrombocytopenia. However, of those with “vaccine-associated ITP,” several patients had relapses necessitating further therapy. Given the variability in presentations that have been observed at our center and the fact that it is as of yet unclear if VITT and “vaccine-associated ITP” are a spectrum of pathology, follow-up in a thrombosis clinic or with a hematologist with a special interest would be most appropriate to manage diagnostic uncertainty and to provide advice when considering further vaccinations against COVID-19.

In summary, the cases presented at our center have demonstrated the heterogeneity that exists in post-COVID-19 vaccine-related immune-mediated complications. Further data collected going forward will aid in the understanding of whether VITT and “vaccine-associated ITP” exist on a “spectrum” or are two distinct entities.

## Figures and Tables

**Table 1:** A summary of the patients included in this case series.

Cases	Diagnosis	Presenting features	Laboratory findings	Management	Platelet recovery time	Outcome
Case 1	Definite VITT^2^	Headaches and critical limb ischemia	Platelet count: 16 × 10^9^/L. D-dimer: 36.00 ug/ml. fibrinogen: 2.6 g/L.Anti-PF4 antibodies: 0.285	IVIg x2 Dexamethasone Argatroban IVI Surgical intervention Aspirin and rivaroxaban postoperatively	6 days	Transfemoral above-knee amputation. Anti-PF4 antibodies were not detected at 28 days

Case 2	Pre VITT/Probable VITT^2^	Headaches and myalgia	Platelet count: 20 × 10^9^/L. D-dimer: 54.30 ug/ml. fibrinogen: 1.3 g/L. Anti-PF4 antibodies: 0.245	IVIg Fondaparinux 6 months rivaroxaban	7 days	6 months rivaroxaban. Anti-PF4 antibodies were not detected at 35 days

Case 3	Vaccine-associated ITP	Mucocutaneous bleeding	Platelet count: 5 × 10^9^/L. D-dimer: 1.03 ug/ml. Anti-PF4 antibodies: 0.247	IVIg Dexamethasone Prednisolone Eltrombopag	No full recovery of platelets when case series written	Continuous prednisolone and eltrombopag

Case 4	Vaccine-associated ITP	Mucocutaneous bleeding	Platelet count: 1 × 10^9^/L. D-dimer: 1.87 ug/ml. Anti-PF4 antibodies: 0.285	IVIg Dexamethasone Prednisolone Eltrombopag	No full recovery of platelets when case series written	Weaning regime of prednisolone. Eltrombopag was discontinued

Case 5	Vaccine-associated ITP	Headaches and mucocutaneous bleeding	Platelet count: 26 × 10^9^/L. D-dimer: *not elevated*. Anti-PF4 antibodies: 0.734	IVIg Dexamethasone Prednisolone	8 days (relapsed on day 15)	Weaning regime of prednisolone

Case 6	Vaccine-associated ITP	Petechiae	Platelet count: 16 × 10^9^/L. D-dimer: *not elevated*. Anti-PF4 antibodies: 0.070	IVIg Dexamethasone	7 days	Full recovery; off treatment

Case 7	Vaccine-associated ITP	Petechiae and mucocutaneous bleeding	Platelet count: 1 × 10^9^/L. D-dimer: *not elevated*. Anti-PF4 antibodies: 0.172	IVIg Dexamethasone 2^nd^ dose IVIg	28 days	Full recovery; off treatment

## Data Availability

The data supporting the findings of this report are included within the article.
